# Do eye diseases increase the risk of arthritis in the elderly population?

**DOI:** 10.18632/aging.203122

**Published:** 2021-06-10

**Authors:** Wenyi Jin, Qian Yao, Zilin Liu, Wenli Cao, Yubiao Zhang, Zhifei Che, Hao Peng

**Affiliations:** 1 Department of Orthopedics, Renmin Hospital of Wuhan University, Wuchang 430060, Wuhan, China; 2 Department of Clinical Laboratory, Renmin Hospital of Wuhan University, Wuchang 430060, Wuhan, China; 3 Department of Ophthalmology, Renmin Hospital of Wuhan University, Wuchang 430060, Wuhan, China; 4 Department of Urology, Renmin Hospital of Changyuan City, Changyuan 450000, Henan Province, China

**Keywords:** eye diseases, cataracts, glaucoma, arthritis

## Abstract

There are very few longitudinal studies which have previously conducted an investigation into whether eye diseases are a risk for arthritis, and how this occurs. The study employed a variety of machine-learning algorithms, including random forest for investigating the risks, and to elucidate these underlying mechanisms by focusing on five aspects containing 389 characterized variables (mental health and wellbeing; physical health; disability, functional impairment and helpers; health behavior; and health measures). The study population included 8,423 individuals. Cataracts, glaucoma, and other eye diseases increase the likelihood of arthritis after two years by 131.8% (odds ratio (OR)=2.318, 95% confidence interval: 1.748 to 3.038), 123.1% (OR=2.231, 1.306 to 3.626), and 91.1% (OR=1.911, 1.501 to 2.415). Random forest corroborated that cataract contributes the most to arthritis risks after two years, followed by other eye diseases and glaucoma (mean Gini-index: 5.20, 2.11, 1.31). It is of note that the potential mechanisms of cataract-induced arthritis risk were elucidated extensively. The control domains of life quality, negative aging self-perceptions, mobility (steadiness, physical limitations, and muscle strength) and memory impairments, and sleep quality mediated the relationship between cataracts and arthritis significantly. Furthermore, different eye diseases affected osteoarthritis, rheumatoid arthritis, and other arthritis to varying degrees. Eye diseases increased the risk of arthritis, whereby cataracts were the most significant. Interventions which target these discovered mechanisms may be the preferred levers for reducing cataract-related arthritis risk.

## INTRODUCTION

Human life expectancy has increased worldwide at an unprecedented rate during the last two centuries, and age is no longer potentially determined by natural selection [1, 2]. The aging population has escalated the global burden of diseases, making the exploration of the reduction of the length and severity of late-life morbidity a major goal for future civilized societies [2]. Arthritis, including osteoarthritis and rheumatoid arthritis, remains a major blight among elder adults and is deemed to be a significant global public health challenge [3–5]. Recent studies on global disease burden have suggested that the estimated prevalence of arthritis worldwide is accelerating, and that age augments this [4, 5]. The global age-standardized point prevalence of knee osteoarthritis is estimated as being 3.7%, increasing to 14% in those aged 65 and over. Rheumatoid arthritis is also more common in elderly people and its global age-standardized point prevalence has reached 1% for those aged 75 years and older [6, 7]. These debilitating joint diseases are a leading source of disability, pain, and socioeconomic cost all over the world. In developed countries, the socioeconomic burden caused by osteoarthritis accounts for 1.0% to 2.5% of gross domestic product [8, 9]. This sobering and salient evidence proves how important the exploration of factors affecting arthritis is for the control or prevention of such diseases.

Certain types of arthritis, including rheumatoid arthritis and juvenile idiopathic arthritis, are universally acknowledged to regularly lead to cataracts or other eye conditions [10, 11]. However, to the best of our knowledge, very little research has ever investigated whether eye diseases increase the risk of arthritis or the ways in which it can occur, particularly among the elderly. Eye diseases, including cataracts and glaucoma, are quite prevalent among the older population and pose a threat to their quality of life [3]. For example, cataracts are a reversible cause of blindness in those aged 50 and above, and together with uncorrected refractive errors, they account for 55% of cases of blindness and 77% of cases of visual impairment [12]. Due to the high prevalence of eye diseases and the resulting functional impairment or comorbidities of the elderly population, an investigation into whether the risk of arthritis varies based on exposure to eye diseases and the underlying mechanisms of this risk is warranted.

The study provides the first report quantifying the effects of various eye diseases on the developmental process of arthritis and identifies cataracts as being a major contributor to the increased risk of arthritis. It is notable that the underlying mechanisms of cataracts leading to arthritis are clarified and can assist clinicians in the application of appropriate interventions for the reduction of arthritis risk among eye patients. The study focuses on five aspects containing 389 characterized variables in order to elucidate the existence of underlying mechanisms in the relationship between cataracts and arthritis. These five aspects are mental health and wellbeing; physical health; disability, functional impairment and helpers; health behavior; and health measures.

## MATERIALS AND METHODS

STROBE (Strengthening the Reporting of Observational Studies in Epidemiology) guidelines were followed for this study [13].

### Participants

Data was obtained by The Irish Longitudinal Study on Ageing (TILDA), a longitudinal study with two waves of population-representative data from Irish participants aged 50 and over who were sampled using a geographic cluster-based RANSAM sampling system [14, 15]. The study targeted individuals aged 50 and over who lived in the Republic of Ireland in residential accommodation (excluding nursing homes and other similar institutions). Full informed consent was provided by a total of 8,504 participants, and Trinity College Dublin Research Ethics Committee granted ethical approval of this.

Between October 2009 and February 2011, participants were contacted for a Wave 1 interview [16], and Wave 2 interviews were conducted between April 2012 and January 2013 [17]. The response rate adjusted for eligibility was 62.0%. Participants were excluded for incomplete covariate data, which resulted in a sample size of 8,423 for cross-sectional analyses. In the data pre-processing of baseline, participants (n = 2589) with missing data for covariates of CHOL, LDL, HDL, and TRIG, and participants (n = 2337) were missing BMI covariate data. Considering that the elimination of large censored data of covariates can create a bias and reduce the sample size, k-nearest neighbor imputation was used to deal with the incomplete covariates [18]. Participants who reported arthritis at baseline (n = 1927) or lost to follow-up at Wave 2 (n = 1261) were excluded from longitudinal analyses, which resulted in a sample size of 5,235.

### Measures

Demographic data for gender, age, smoking status, and marital status were collected in Wave 1 interviews.

Eye diseases and arthritis were investigated during Waves 1 and 2. The senior ophthalmologists and orthopedic specialists diagnosed and collected clinical parameters, including cataracts, glaucoma, other eye diseases, arthritis and its subtypes of osteoarthritis, rheumatoid arthritis, and other types of arthritis in Wave 1, and reaffirmed whether participants had arthritis in Wave 2. Other eye diseases refers to eye diseases other than cataracts and glaucoma.

Mental health and wellbeing were evaluated using scales and questions in Wave 1. The Center for Epidemiologic Studies Depression Scale [19], Hospital Anxiety and Depression Scale [20], CASP-19 Scale [21], Aging Perceptions Questionnaire [22], Penn State Worry Questionnaire [23], UCLA Loneliness Scale [24], and Computer-aided personal interview questionnaire were utilized and 109 variables were selected to characterize mental health and wellbeing.

Physical health, disability, functional impairment and helpers were assessed using a series of tests and questionnaires in Wave 1. Activities of Daily Living, Instrumental Activities of Daily Living, diseases, disability etc., were characterized by 168 variables (125 variables from the physical health aspect and 33 from the disability, functional impairment and helpers aspect).

Health measures were conducted at fixed health assessment centers in Dublin or Cork for the collection of objective measures of physical health. Respondents who were unable or unwilling to attend a health assessment center were given the option of participating in a shorter, home-based assessment. Some measurements, including visual acuity, could only be measured at the health centers. A total of 96 variables were included in the baseline, including height, weight, body mass index (BMI), waist and hip circumference, grip strength, visual acuity, blood pressure, blood lipids, and other indicators, Mini-mental state examination (MMSE), picture memory test, and visual reasoning were employed for this purpose.

Health behaviors were characterized by the International Physical Activity Questionnaire in Wave 1, which included a total of 16 derived variables.

Covariates were chosen based on theoretical, practical, and previous empirical evidence associated with arthritis [25–27]. Age, gender, smoking status, marital status, osteoporosis, depression, time and degree of physical activity, BMI, blood pressure, TRIG, LDL, HDL, and CHOL were all included.

All reported variables in these five aspects can be viewed at tilda.tcd.ie.

### Statistics

Binomial logistic regression quantified the associations (i.e. odds ratios) between eye diseases (cataracts, glaucoma, and other eye diseases) and arthritis (all arthritis, osteoarthritis, rheumatoid arthritis, other types of arthritis) at the baseline and in Wave 2. A Hosmer-Lemeshow test was subsequently conducted and Nagelkerke R^2^ was calculated in order to evaluate the models’ goodness of fit [28]. Likelihood ratio tests assessed the significance of the covariates [29].

Spearman correlation analyses were performed to gain further evidence of the relationship between eye diseases and arthritis. The contribution and importance of each subtype of eye diseases in relation to arthritis were quantified using mean Gini-index which was derived from a random forest algorithm (1,000 decision trees), which is a reliable machine-learning method [30].

A series of mediation models were used to reveal the potential mechanisms which mediated the effects on the outcomes [31]. As previously mentioned, there were five aspects of potential mechanisms which focused on: mental health and wellbeing; physical health; disability, functional impairment and helpers; indicators from health measures; and health behaviors. 389 derived variables were included as candidate mediators for mediation analyses where the potential mechanisms of the remaining direct effect of eye diseases and the indirect/mediated effects of potential mediators could be detected to the greatest extent. Censored data for each candidate mediator in a specific mediation model was eliminated independently in order to circumvent any potential bias and reduction to the sample size. Further details relating to measures and analyses are available upon request.

To perform stratified analyses and gender-related grouped analyses, Kruskal–Wallis one-way ANOVA test (H test, a pairwise comparison using Dwass-Steel-Critchlow-Fligner test, p-value adjustment using the Benjamini and Hochberg method), and binomial logistic regression were used for association detection and quantification. It is notable that the effect size was calculated based on different analyses to supplement the adjusted p-value for the quantification of significant difference.

Details of the software and packages that were used to conduct statistical analyses: R (version 3.6.3) was used for statistical analyses. Packages used in the study: Hosmer-Lemeshow test (ResourceSelection package); likelihood ratio test (lmtest package); Wilcoxon test, Kruskal–Wallis one-way ANOVA test, Dwass-Steel-Critchlow-Fligner test, Benjamini and Hochberg method was used for p-value adjustment, and corresponding effect sizes calculation (ggstatsplot package); Random forest algorithm (randomForest package); mediation analyses (mediation package).

## RESULTS

### Demographics of participants on the baseline

The baseline characteristics of the Irish participants aged 50 and over are shown in Table 1. All eye diseases demonstrated significant difference between the no arthritis and arthritis groups: cataracts (p<0.001, *V*=0.113, 95% CI: 0.092 to 0.133, small effect size); glaucoma (p=0.003, *V*=0.031, 95% CI: 0.006 to 0.056, small effect size); and other eye diseases (p<0.001, *V*= 0.144, 95% CI: 0.121 to 0.167, small effect size).

**Table 1:** Demographics of Irish participants on baseline.

**Parameters**		**No arthritis**	**Arthritis**	***p***	**Effect size (95%CI)**
		**(n = 6151)**	**(n = 2272)**		
**Age (year)**		60.00 (54.00-68.00)	67.00 (59.00-74.25)	< 0.001	*r* = 0.243 (0.224-0.262)
**Gender**	**Male**	2943 (78.67)	798 (21.33)	< 0.001	*V* = 0.113 (0.092-0.133)
	**Female**	3208 (68.52)	1474 (31.48)		
**Cataracts**	**With**	484 (54.50)	404 (45.50)	< 0.001	*V* = 0.143 (0.117-0.167)
	**Without**	5667 (75.21)	1868 (24.79)		
**Glaucoma**	**With**	124 (63.59)	71 (36.41)	0.003	*V* = 0.031 (0.006-0.056)
	**Without**	6027 (73.25)	2201 (26.75)		
**Other eyesdiseases**	**With**	787 (58.38)	561 (41.62)	< 0.001	*V* = 0.144 (0.121-0.167)
	**Without**	5364 (75.82)	1711 (24.18)		

Unless otherwise specified, all skewed continuous variables were described by “median (interquartile range)” and all categorical variables by “frequency (constituent ratio (%))”.

Abbreviation: Confidence interval (CI); Cohen’s *r* (*r*); Cramer’s *V* (*V*).

Despite the fact that all eye diseases showed statistical significance, the effect sizes were dissimilar, which indicates that cataracts were the most important, followed by other eye diseases and glaucoma, in terms of their contribution to the outcomes.

### Cross-sectional and longitudinal results

Binomial logistic regression allowing association quantification (i.e. odds ratios) was performed to evaluate the effects of eye diseases on the development of arthritis.

For the cross-sectional studies, the prevalence of arthritis was 26.97% (n=2272). The crude models attested that cataracts, glaucoma, and other eye diseases had a significant association with 153.2% (OR= 2.532, 95%CI: 2.196 to 2.918, *p*<0.001), 56.8% (OR=1.568, 95%CI: 1.162 to 2.101, *p*<0.01), and 123.5% (OR=2.235, 95%CI: 1.979 to 2.522, *p*<0.001) higher odds of arthritis, respectively. Following adjustments made for a series of covariables, ORs as the quantitative indicators of associations between cataracts, glaucoma, other eye diseases and arthritis, displayed fluctuations but generally remained stable (Table 2), thereby confirming the robustness of the aforementioned discovered associations. With the benefit of the refined questionnaires, association assessments were conducted between eye diseases and arthritis subtypes. As the resultant data from crude models shows in Table 2, cataracts increased the incidence of osteoarthritis, rheumatoid arthritis, and other types of arthritis by 117.0% (OR=2.170, 95%CI: 1.818 to 2.579, *p*<0.001), 71.2% (OR=1.712, 95%CI: 1.374 to 2.115, *p*<0.001), and 80.0% (OR=1.800, 95%CI: 1.818 to 2.579, *p*<0.001), respectively. At the same time, the developmental processes of osteoarthritis and rheumatoid arthritis were unaffected by glaucoma, which significantly augmented the risk of other types of arthritis by 128.9% (OR=2.289, 95%CI: 1.225 to 3.918, *p*<0.01). Other eye diseases also showed a significant association with 82.5% (OR=1.825, 95%CI: 1.560-2.129, *p*<0.001), 74.3% (OR=1.743, 95%CI: 1.445-2.091, *p*<0.001), and 88.4% (OR=1.884, 95%CI: 1.416-2.486, *p*<0.001) higher odds of osteoarthritis, rheumatoid arthritis, and other types of arthritis, respectively. The robustness of these associations was corroborated by adjusting a series of covariables, and the associations of reporting significant differences remained of statistical significance (Table 2).

**Table 2:** Odds ratios (OR) and 95% confidence intervals (CI) derived from binominal logistic regression analyses as indicators of association between eyes diseases and arthritis at Wave 1 and 2.

**Eyes diseases**		**Cases/participants**	**Outcomes**	**Crude model** **OR (95% CI)**	**Model 1** **OR (95% CI)**	**Model 2** **OR (95% CI)**	**Model 3** **OR (95% CI)**
Cataracts	*Without*	7535/8423	*^1^Arthritis (ALL)*	2.532	2.459	2.262	2.000
	*With*	888/8423		(2.196-2.918)	(2.13-2.836)	(1.955-2.616)	(1.717-2.327)
				***	***	***	***
Glaucoma	*Without*	8228/8423		1.568	1.589	1.498	1.368
	*With*	195/8423		(1.162-2.101)	(1.174-2.134)	(1.104-2.017)	(0.999-1.859)
				**	**	**	**
Other eyes	*Without*	7075/8423		2.235	2.184	2.039	1.826
diseases	*With*	1348/8423		(1.979-2.522)	(1.933-2.467)	(1.801-2.307)	(1.605-2.077)
				***	***	***	***
Cataracts	*Without*	7535/8423	*^1^Arthritis (OS)*	2.170	2.058	1.993	1.770
	*With*	888/8423		(1.818-2.579)	(1.721-2.452)	(1.661-2.383)	(1.465-2.132)
				***	***	***	***
Glaucoma	*Without*	8228/8423		1.394	1.419	1.378	1.270
	*With*	195/8423		(0.933-2.018)	(0.946-2.066)	(0.917-2.008)	(0.84-1.866)
						
Other eyes	*Without*	7075/8423		1.825	1.748	1.697	1.509
diseases	*With*	1348/8423		(1.560-2.129)	(1.491-2.043)	(1.445-1.988)	(1.277-1.778)
				***	***	***	***
Cataracts	*Without*	7535/8423	*^1^Arthritis (RA)*	1.712	1.691	1.546	1.331
	*With*	888/8423		(1.374-2.115)	(1.357-2.091)	(1.235-1.919)	(1.057-1.664)
				***	***	***	*
Glaucoma	*Without*	8228/8423		1.346	1.349	1.269	1.155
	*With*	195/8423		(0.826-2.081)	(0.828-2.086)	(0.778-1.966)	(0.703-1.805)
						
Other eyes	*Without*	7075/8423		1.743	1.727	1.607	1.428
diseases	*With*	1348/8423		(1.445-2.091)	(1.432-2.073)	(1.329-1.936)	(1.174-1.73)
				***	***	***	***
Cataracts	*Without*	7535/8423	*^1^Arthritis (Other)*	1.800	1.781	1.620	1.494
	*With*	888/8423		(1.281-2.476)	(1.267-2.452)	(1.144-2.246)	(1.047-2.089)
				***	***	**	*
Glaucoma	*Without*	8228/8423		2.289	2.294	2.173	2.143
	*With*	195/8423		(1.225-3.918)	(1.227-3.927)	(1.161-3.728)	(1.139-3.703)
				**	**	**	*
Other eyes	*Without*	7075/8423		1.884	1.869	1.739	1.636
diseases	*With*	1348/8423		(1.416-2.486)	(1.399-2.467)	(1.296-2.308)	(1.211-2.186)
				***	***	***	**
Cataracts	*Without*	4850/5235	*^2^Arthritis (ALL)*	2.318	2.308	2.199	2.002
	*With*	385/5235		(1.748-3.038)	(1.74-3.025)	(1.651-2.894)	(1.495-2.651)
				***	***	***	***
Glaucoma	*Without*	5134/5235		2.231	2.238	2.150	1.917
	*With*	101/5235		(1.306-3.626)	(1.309-3.639)	(1.255-3.502)	(1.111-3.147)
				***	**	**	*
Other eyes	*Without*	4597/5235		1.911	1.901	1.825	1.654
diseases	*With*	638/5235		(1.501-2.415)	(1.492-2.401)	(1.429-2.313)	(1.289-2.106)
				***	***	***	***

Model 1 adjusted for age, sex. Model 2 adjusted for Model 1 and smoking status, marital status. Model 3 adjusted for Model 2 and psychiatric problems (such as depression and anxiety), high blood pressure, osteoporosis, physical activity (time), physical activity (degree), BMI, TRIG, LDL, HDL, CHOL.

Abbreviations: *^1^Arthritis (ALL)*, Arthritis (ALL) at Wave 1; *^1^Arthritis (OS)*, Arthritis (osteoarthritis) at Wave 1; *^1^Arthritis (RA)*, Arthritis (rheumatoid arthritis) at Wave 1; *^2^Arthritis (ALL)*, Arthritis (ALL) at Wave 2; ***, p-value < 0.001; **, p-value < 0.01; *, p-value < 0.05.

For the longitudinal studies, the prevalence of probable arthritis was 9.59% (n=502). The current crude models suggested that cataracts, glaucoma, and other eye diseases increased the risk of arthritis after two years by 131.8% (OR=2.318, 95%CI: 1.748 to 3.038, *p*<0.001), 123.1% (OR=2.231, 95%CI: 1.306 to 3.626, *p*<0.001), and 91.1% (OR=1.911, 95%CI: 1.501 to 2.415, *p*<0.001), respectively (Table 2). As anticipated, these associations remained significant statistically when the crude models were adjusted for covariables.

In addition, following prudent consideration of the results of the goodness-of-fit test and the likelihood ratio test (Supplementary Table 1), model 1, model 2, and model 3 were deemed to be different during sensitivity analyses, which thereby validated the robustness of the detected associations.

### Contributions of eye diseases to arthritis

**Figure 1:**
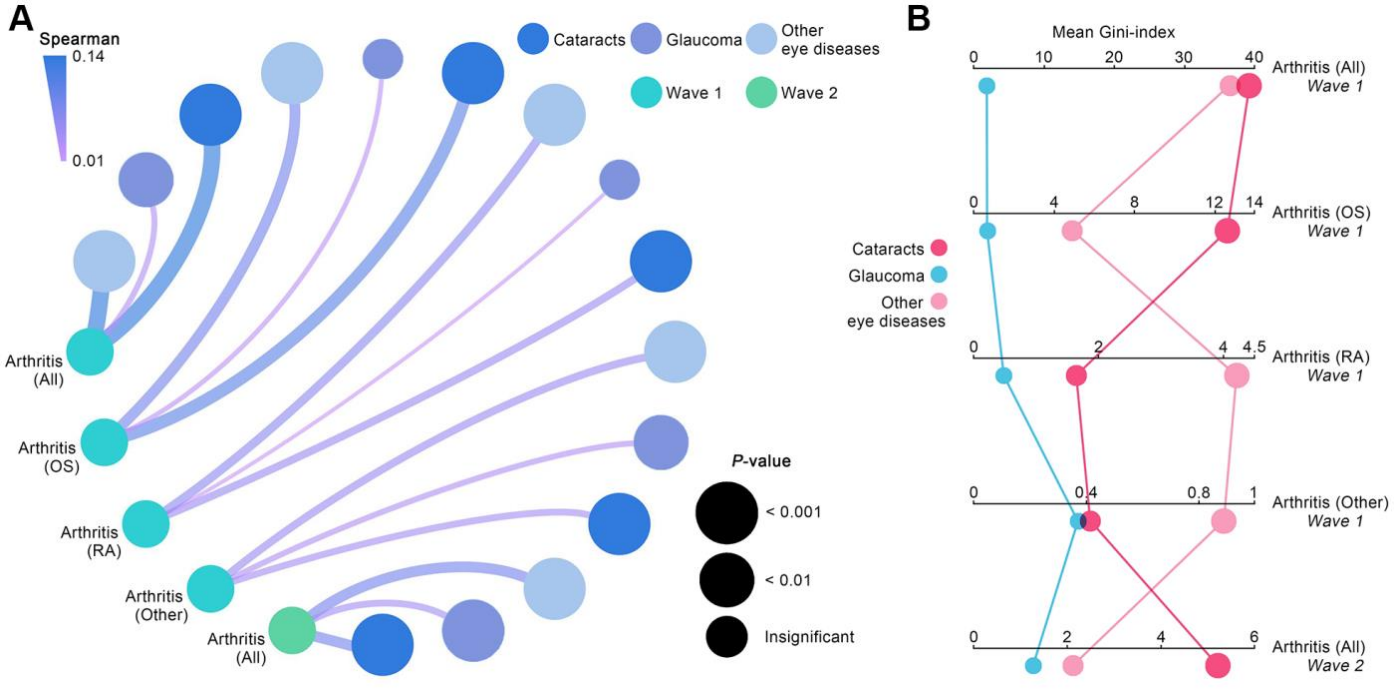
**Contributions of eye diseases to arthritis.** (**A**) Spearman coefficients quantified the correlations between various eye diseases and arthritis, including its subtypes. (**B**) Mean Gini-index calculated from random forest algorithm quantified the degree of contribution of each eye disease to arthritis.

Correlations between each eye disease and arthritis, including all subtypes, were reaffirmed in Waves 1 and 2. The resultant data from the correlation analyses matched the derived results from the aforementioned binomial logistic regression (Figure 1A). Baseline analyses conducted using Spearman coefficient confirmed that all eye disease types had a significant correlation with arthritis (all *p*<0.01). For analyses of arthritis subtypes, cataracts and other eye diseases showed a significant association with all arthritis subtypes (all *p*<0.01), and glaucoma only affected other types of arthritis (*p*<0.01). Wave 2 analyses for correlations further corroborated that all eye diseases had a significant association to a higher probability of arthritis.

In order to quantify each eye disease’s contribution to arthritis incidence, mean Gini-index derived from random forest algorithm was used. As the resultant data in Figure 1B shows, cataracts were the most valuable impactor on the developmental process of arthritis in Waves 1 and 2, followed by other eye diseases and glaucoma (mean Gini-index of cataracts, glaucoma, and other eye diseases: 39.29, 1.79, 36.74 in Wave 1; 5.20, 2.11, 1.31 in Wave 2). For baseline analyses aimed at arthritis subtypes, cataracts were discovered to be the most significant contributor to osteoarthritis development (mean Gini-index = 12.58), while other eye diseases made a much higher contribution to rheumatoid arthritis (mean Gini-index = 4.22) and other types of arthritis (mean Gini-index = 0.89) than cataracts and glaucoma.

### Underlying mechanisms mediating the increased risk of arthritis in Wave 2

Among all the eye diseases, the study has proven that cataracts have the most significant impact on increased arthritis risk. Therefore, further investigation of the underlying mechanisms between cataracts and arthritis was conducted. As previously mentioned, in order to extensively reveal these underlying mechanisms, 389 variables from five aspects characterizing elderly adults’ health were investigated using mediation analyses for the detection of potential mediators. This method was based on the previous study [32], and the five aspects of the study were: mental health and wellbeing; physical health; disability, functional impairment and helpers; indicators from health measures; and health behaviors.

**Figure 2:**
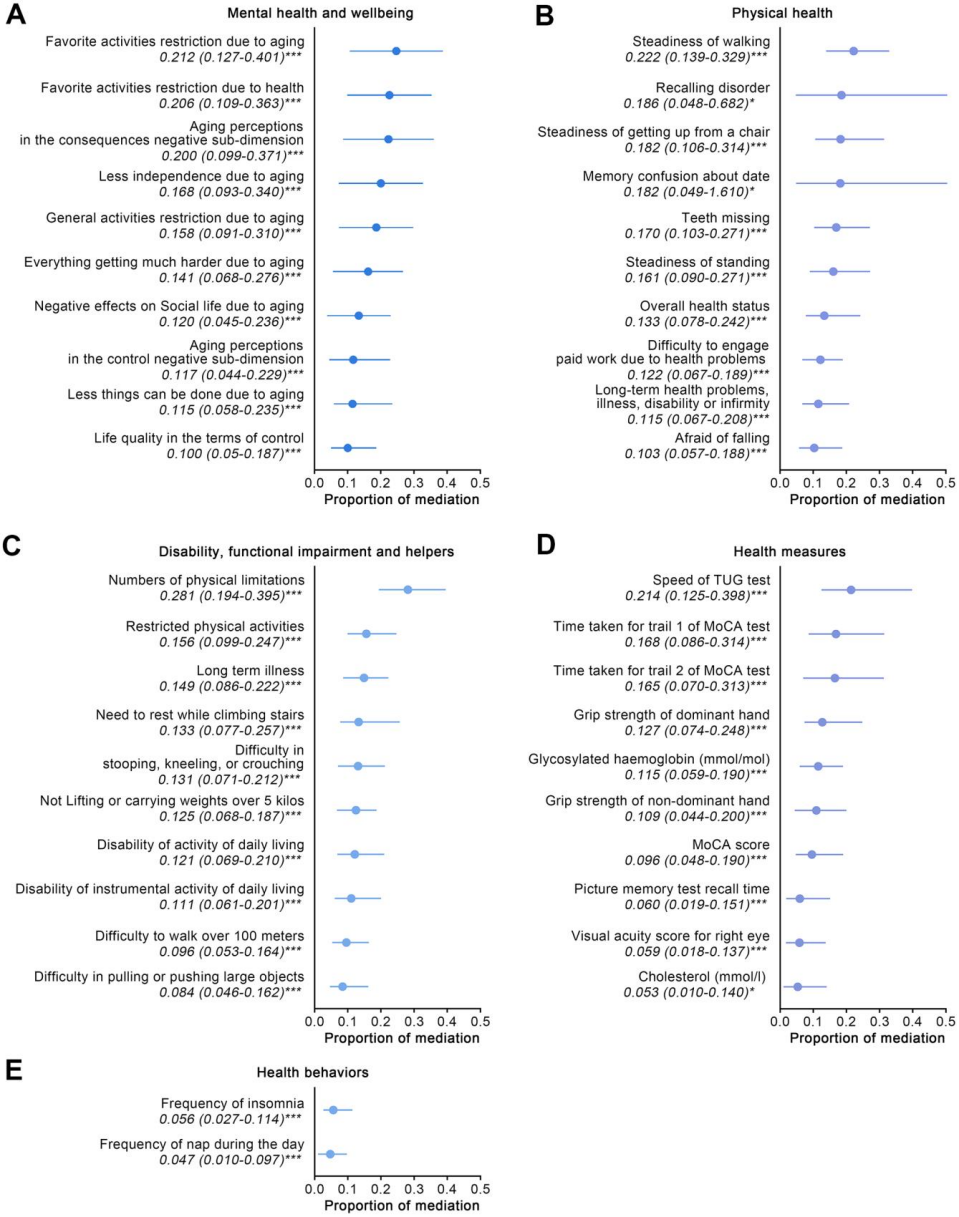
**The effects of mediators which characterized five aspects of participants aged 50 and over on Wave 2 arthritis.** (**A**–**E**) For the aspects of mental health and wellbeing; physical health; disability, functional impairment and helpers; health behavior; and health measures, quantified proportions of mediation of the top 10 significant mediators for each aspect are shown. Abbreviations: TUG: timed up and go; MoCA: Montreal Cognitive Assessment. ***: p-value < 0.001; **: p-value < 0.01; *: p-value < 0.05.

In the mental health and wellbeing aspect, 29 variables significantly mediated associations between cataracts and arthritis from 109 derived variables, and the top 10 mediators ranked by proportion of mediation are shown in Figure 2A. Most significant variables were derived from the CASP-19 scale and Aging Perceptions questionnaire, showing that worse control domain of life quality, consequences, and control negative sub-dimensions of aging perceptions were the remarkable mediators for the relationship between cataracts and arthritis.

With mediation analyses for the physical health aspect, 33 variables were shown to be significant mediators from 125 variables, and the top 10 mediators are displayed in Figure 2B. Steadiness-related variables impacted the aforementioned relationships most significantly (proportion of mediation: 0.222, 95% CI: 0.139 to 0.329, of steadiness of walking), followed by memory issues, teeth missing, and long-term health problems, illness, disability or infirmity. It is notable that cataract surgery’s potential effect on arthritis risk was also tested and the resultant data suggested that there are no mediation effects (*p* = 0.630). Further analyses aimed at the detection of potential differences of arthritis prevalence in cataract participants also failed to achieve statistical significance (with arthritis *vs.* without arthritis: 19% *vs.* 17%, *p* = 0.623).

In the aspect of disability, functional impairment and helpers, 22 variables demonstrated significant effects on the outcomes from the 33 candidate mediators, and the top 10 mediators are shown in Figure 2C. Greater physical limitations was the main mediator of associations between cataracts and arthritis (proportion of mediation: 0.281, 95% CI: 0.194 to 0.395), followed by long-term illness (proportion of mediation: 0.149, 95% CI: 0.086 to 0.222).

For the health measures aspect, 31 indicators corroborated the significant mediators affecting outcomes from 96 variables, and Figure 2D displays the top 10 mediators. Functional mobility impairment was shown to have the most significant impact on outcomes (proportion of mediation: 0.214, 95% CI: 0.125 to 0.398, of speed of timed up and go test), followed by indicators pertaining to memory and muscle strength (grip strength of dominant and non-dominant hand), and glycosylated hemoglobin (HbA1c), CHOL.

In aspect of the health behaviors, only two mediators were statistically significant, from the 16 included candidate mediators (Figure 2E). The resultant data showed that sleep quality, including insomnia and poor spirit during the daytime, mediated the associations between cataracts and arthritis.

### Gender-related differences

The present study further investigated whether gender difference had an effect on the risk of arthritis in Wave 2. When only subjects suffering from cataracts, glaucoma, or other eye diseases were included in the analyses, none of these diseases were reported to be of any significant statistical difference between males and females in terms of arthritis prevalence in Wave 2 (Supplementary Figure 1).

## DISCUSSION

This study which uses data from a population-representative sample of elderly people living in the same context is, to our knowledge, the first attempt to investigate whether different eye diseases increase arthritis risk and how this occurs. With the benefit of extensive detection of underlying mechanisms regarding such risk offered by the current study, clinicians could choose an appropriate intervention for older cataract patients in order to reduce arthritis risk.

The current study discovered that those aged 50 and over suffering from cataracts, glaucoma, or other eye diseases increased the probability of arthritis after two years by 131.8% (OR=2.318, 95%CI: 1.748 to 3.038, *p*<0.001), 123.1% (OR=2.231, 95%CI: 1.306 to 3.626, *p*<0.001), and 91.1% (OR=1.911, 95%CI: 1.501 to 2.415, *p*<0.001), respectively. The findings were reaffirmed using correlation analyses and random forest algorithm, and cataracts were identified as the most valuable contributor to an augmented risk of arthritis (mean Gini-index of 5.20 in Wave 2), followed by other eye diseases and glaucoma.

Previous research basically claimed that ocular comorbidities were resulted by joint diseases [10, 11, 33–35], including juvenile idiopathic arthritis-induced cataracts, and psoriatic arthritis-associated eye comorbidities. However, few studies have reported whether eye diseases increase the probability of arthritis. A study on data from the Agricultural Health Study (AHS) offered preliminary evidence that cataracts are a risk factor for the development of rheumatoid arthritis [36], which is similar to our resultant data for rheumatoid arthritis and cataracts where cataracts were shown to increase the risk of rheumatoid arthritis by 71.2% (OR=1.712, 95%CI: 1.374 to 2.115) in Wave 1. However, insufficient sample size is a limitation for the strength of the evidence of the data which was reported in the AHS study [36], and the study only focused on the relationship between rheumatoid arthritis and cataracts. The association that was discovered between cataracts and rheumatoid arthritis showed the common etiologies between the diseases. Despite cataracts not generally being considered to be an immune-related disease, their appearance has increased among those with autoimmune diseases (such as rheumatoid arthritis) [36]. A unifying role of the oxidative stress between cataracts and rheumatoid arthritis has also been suggested [37]. The baseline analyses further elucidated the effects of a number of eye diseases on the development of arthritis subtypes, including osteoarthritis, rheumatoid arthritis, and other types of arthritis. These findings provide implicated instruction for the clinical treatment of the elderly suffering from eye diseases to help reduce arthritis risk and improve quality of life.

The importance of this study lies in the completion of extensive detection of potential mechanisms that exist between cataracts and arthritis in Wave 2. Results from analyses regarding mental health and wellbeing showed that a worse control domain of life quality, consequences and control negative sub-dimensions of aging perceptions mediated the effect of cataracts on arthritis. The coexistence of adverse mental health and arthritis has long been recognized [27, 38, 39]. A longitudinal study on regional cohorts suggested that depression increases the risk of rheumatoid arthritis by 65% [40]. In other studies, controlling depressive symptoms remised rheumatoid arthritis or reduced rheumatoid arthritis risk [41, 42]. This study’s findings further demonstrated that the implementation of interventions for improving quality of life regarding control domain and maintaining positive self-perceptions of aging, could effectively reduce arthritis risk.

Within our ecological perspective, mobility (steadiness of walking, getting up from a chair, and standing) and memory issues also interpret the association between cataracts and arthritis. The effect of mobility on arthritis risk could be ascribed to local mechanical factors or abnormal loading of the joints [43–46]; memory impairment and arthritis have high comorbidity [47, 48]. As cataracts are a long-term illness which affects mobility, further examination of whether cataract surgery has an influence on the relationship between cataracts and arthritis. The resultant data was quite illuminating and showed that cataract surgery, as a well-known protective factor for the steadiness of mobility [49], did not mediate increased arthritis risk. Also, no significant difference has been reported in prevalence analyses of cataract sufferers who have undergone or not undergone cataract surgery. Enlightened by this evidence, direct interventions on mobility and memory issues could be a more effective lever for the reduction of arthritis risk in cataract patients.

Regarding the disability, functional impairment and helpers aspect, this study found that physical limitations was the most valuable mediator in the relationship between cataracts and arthritis. The mediation effects were as anticipated, as the restriction of physical activity and functional mobility impairment are considered to be factors which can potentially increase arthritis risk [50–52]. The mediation analyses for the health measures aspect reaffirmed the mediation effects of mobility and memory impairment, while highlighting that muscle strength (grip strength of the dominant and non-dominant hand) mediated the increased risk of arthritis. Muscle strength partly reflected mobility impairment (including physical limitations and steadiness), and previous research reported a loss of muscle strength as being an arthritis risk factor [52]. Further mediation analyses of the health behavior aspect discovered that sleep quality (insomnia and poor daytime spirits) affected the association between cataracts and arthritis. These results emphasized the role played by sleep quality in arthritis development.

The limitations of this study must be acknowledged. With the design restrictions of the Wave 2 questionnaire interviews, a longitudinal associations assessment between different eye diseases and subtypes of arthritis was impossible. Therefore, baseline analyses were performed investigating the effects of different eye diseases on arthritis subtypes, including osteoarthritis, rheumatoid arthritis, and other types of arthritis. The effects of various eye diseases on the development of arthritis were quantified in Wave 2 and the underlying effects of these mechanisms were extensively detected. However, there is a need for further longitudinal studies on eye diseases and arthritis subtypes.

**Figure 3:**
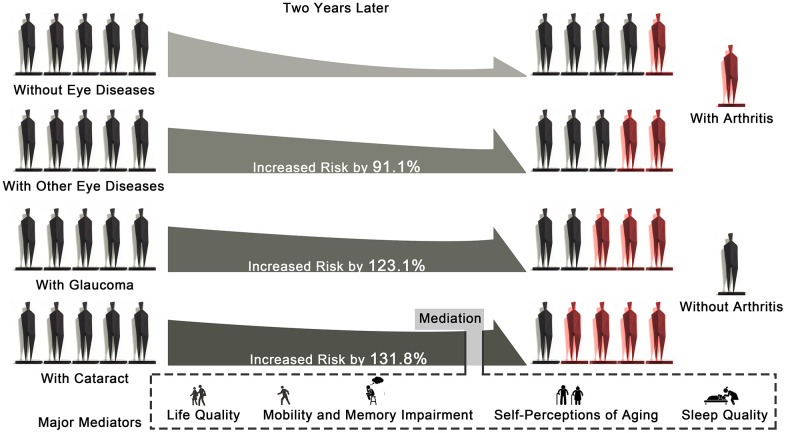
Graphical abstract showing the major findings of the study.

The study discovered and quantified the effects of eye diseases (cataracts, glaucoma, and other eye diseases) on the developmental process of arthritis and its subtypes, including osteoarthritis, rheumatoid arthritis, and other types of arthritis. It further identified cataracts as being the most important contributor to increased arthritis risk in Wave 2. It is notable that by relying on extensive mediation analyses of five aspects containing 389 derived variables, the existence of underlying mechanisms between cataracts and arthritis were clarified in Wave 2 by this study. The findings highlighted the mediation effects of the control domain of life quality, consequences and control negative sub-dimensions of aging perceptions, mobility (steadiness, physical limitations, and muscle strength), memory impairment, and sleep quality on the associations between cataracts and arthritis. These findings can instruct clinicians to consider increased arthritis risk to be a result of eye diseases, in particular cataracts. Interventions that target life quality, self-perceptions of aging, mobility and memory impairment, and sleep quality may be a preferred lever for the reduction of arthritis risk among elderly people with cataracts. Major findings of the present study were exhibited in Figure 3.

### Role of the funder/sponsor

The funding sources played no role in the designing or conducting of this study.

### Availability of data and materials

All data which was generated or analyzed during this study is included in this published article.

## Supplementary Material

Supplementary Figure 1

Supplementary Table 1
