# Supplementary material for: Do eye diseases increase the risk of arthritis in the elderly population?
Source: Aging (Albany NY). 2021 Jun 10;13(11):15580–94. doi: 10.18632/aging.203122 (PMC8221314; doi:10.18632/aging.203122)
Supplement: Supplementary Figure 1 [file aging-13-203122-s001.pdf]

SUPPLEMENTARY FIGURE

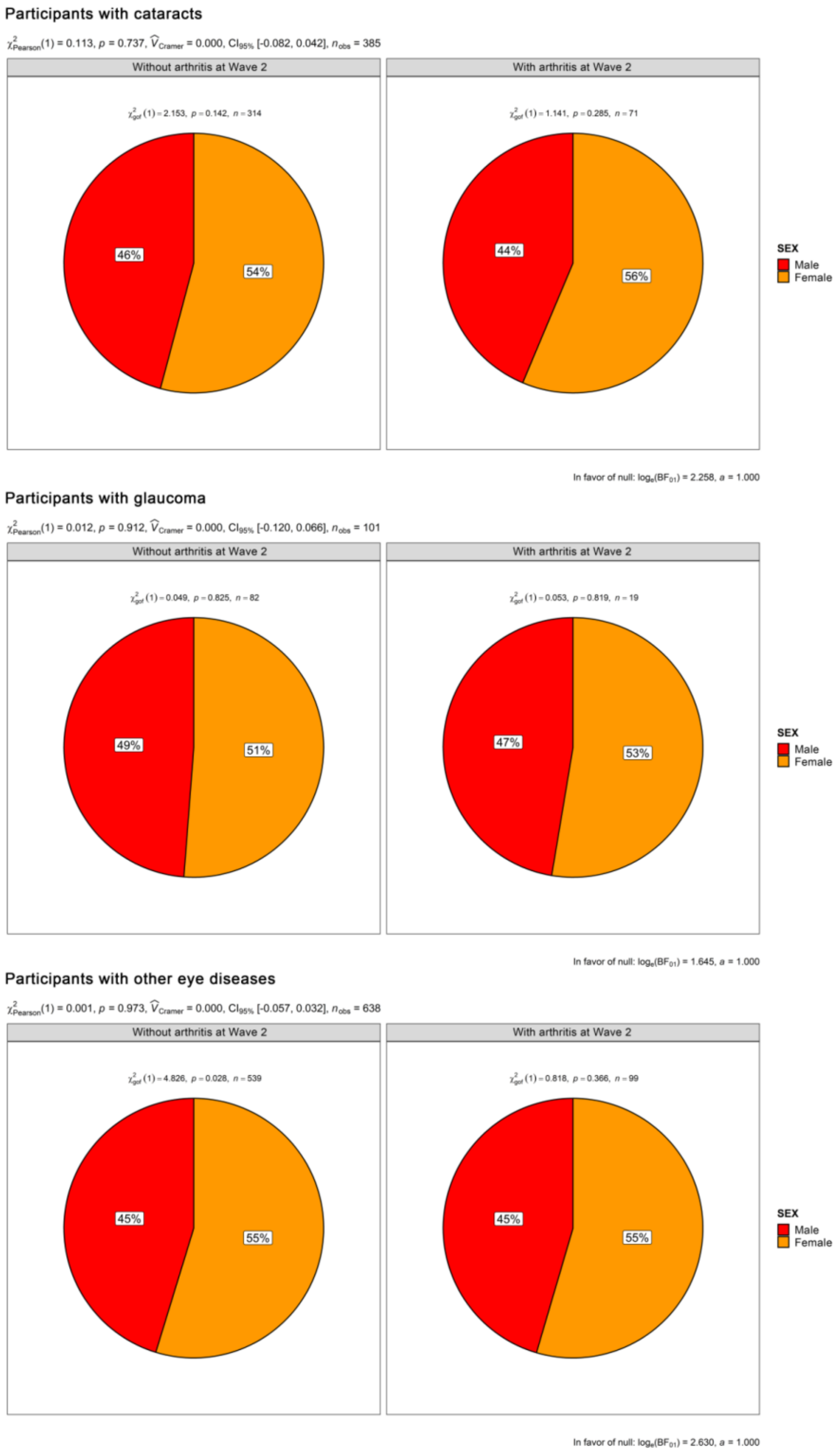

Supplementary Figure 1. Gender-related differences of participants with eyes diseases on Wave 2 arthritis.
