# Supplementary material for: Do eye diseases increase the risk of arthritis in the elderly population?
Source: Aging (Albany NY). 2021 Jun 10;13(11):15580–94. doi: 10.18632/aging.203122 (PMC8221314; doi:10.18632/aging.203122)
Supplement: Supplementary Table 1 [file aging-13-203122-s002.pdf]

SUPPLEMENTARY TABLE

Supplementary Table 1. Hosmer and Lemeshow goodness of fit test and Likelihood ratio test for model 1, 2, and 3 which employed in sensitivity analyses in Wave 1 and 2.

Eye diseases	Outcomes	Hosmer and Lemeshow goodness of fit test			Likelihood ratio test	
		Model 1	Model 2	Model 3	Model 1 vs. Model 2	Model 2 vs. Model 3
Cataracts	[1]*Arthritis (ALL)*	0.877	0.325	0.092	***	***
glaucoma		1.000	0.017	0.000	***	***
Other eye diseases		0.984	0.580	0.400	***	***
Cataracts	[1]*Arthritis (OS)*	0.994	0.070	0.044	ns	***
glaucoma		1.000	0.023	0.066	**	***
Other eye diseases		0.952	0.057	0.155	ns	***
Cataracts	[1]*Arthritis (RA)*	1.000	0.124	0.000	***	***
glaucoma		1.000	0.021	0.001	***	***
Other eye diseases		0.993	0.296	0.000	***	***
Cataracts	[1]*Arthritis (Other)*	0.997	0.135	0.037	*	***
glaucoma		1.000	0.097	0.270	**	***
Other eye diseases		1.000	0.205	0.048	*	***
Cataracts	[2]*Arthritis (ALL)*	1.000	0.992	0.044	**	***
glaucoma		1.000	0.999	0.096	***	***
Other eye diseases		0.997	0.975	0.135	**	***

Abbreviations: [1]*Arthritis (ALL)*, Arthritis (ALL) at Wave 1; [1]*Arthritis (OS)*, Arthritis (osteoarthritis) at Wave 1; [1]*Arthritis (RA)*, Arthritis (rheumatoid arthritis) at Wave 1; [2]*Arthritis (ALL)*, Arthritis (ALL) at Wave 2; ***, p-value < 0.001; **, p-value < 0.01; *, p-value < 0.05; ns, no-significant.
